# Dynamics of the lung microbiome in intensive care patients with chronic obstructive pulmonary disease and community-acquired pneumonia

**DOI:** 10.1038/s41598-020-68100-4

**Published:** 2020-07-06

**Authors:** Wei-Chang Huang, Ming-Feng Wu, Chen-Cheng Huang, Sin-Yin Liu, Hui-Chen Chen, Yih-Yuan Chen, Jeng-Yuan Hsu, Chieh-Chen Huang

**Affiliations:** 1 Department of Life Sciences, National Chung Hsing University, 145 Xingda Rd., South Dist., Taichung, 402 Taiwan; 2 Division of Chest Medicine, Department of Internal Medicine, Taichung Veterans General Hospital, Taichung, 407 Taiwan; 3 Department of Medical Technology, Jen-Teh Junior College of Medicine, Nursing and Management, Miaoli, 350 Taiwan; 4 Department of Industrial Engineering and Enterprise Information, Tunghai University, Taichung, 407 Taiwan; 5 Department of Medical Laboratory Science and Biotechnology, Central Taiwan University of Science and Technology, Taichung, 406 Taiwan; 6 Division of Chest Medicine, Department of Internal Medicine, Taichung Hospital, Ministry of Health and Welfare, Taichung, 403 Taiwan; 7 Department of Biochemical Science and Technology, National Chiayi University, Chia-Yi, 600 Taiwan; 8 Division of Clinical Research, Department of Medical Research, Taichung Veterans General Hospital, Taichung, 407 Taiwan; 9 School of Medicine, China Medical University, Taichung, 404 Taiwan; 10 School of Physical Therapy, Chung-Shan Medical University, Taichung, 402 Taiwan

**Keywords:** Microbiome, Chronic obstructive pulmonary disease

## Abstract

Little is known about the composition and clinical implications of lung microbiome in patients with chronic obstructive pulmonary disease (COPD) and community-acquired pneumonia requiring invasive mechanical ventilation and intensive care unit admission. Therefore, this study aimed to explore the longitudinal changes in microbial airway composition and its variations between COPD patients with different weaning outcomes. Fifty-one endotracheal aspirate samples from 21 participants and 5 saline samples were collected as the patient and control group, respectively. Sequence analysis revealed significant increases and upward trends in the relative abundance of the *Acinetobacter* genus and *Acinetobacter baumannii* complex species in paired comparisons of sampling points and over time, respectively, in patients with failed weaning (p for trend = 0.012 and 0.012, respectively) but not in those with successful weaning (p for trend = 0.335 and 0.426, respectively). Furthermore, significant changes in the composition of the bacterial community were observed in paired comparisons of sampling points in patients with failed weaning compared with those with successful weaning. The alpha diversity did not differ between the patients with different weaning outcomes. These results further the understanding of longitudinal airway microbiome structure analysis and its clinical implications when managing critically ill patients with and without COPD.

## Introduction

Chronic obstructive pulmonary disease (COPD) is a chronic inflammatory disorder caused by the inhalation of tobacco smoke or other irritants. COPD is characterized by persistent respiratory symptoms and airflow limitations, and it is one of the leading causes of morbidity and mortality worldwide^1^. Community-acquired pneumonia (CAP) is one of the most common secondary respiratory infections observed in patients with COPD, due to their impaired lung defense mechanisms^2,3^. When severe, CAP increases the risk of requiring invasive mechanical ventilation (IMV), difficulty in weaning from IMV and mortality^4–7^.

The lung microbiome is emerging as an important research frontier in COPD. To date, the majority of research has focused on changes in the composition of the microbial community, which may be associated with disease progression, exacerbation events, specifically administered treatments, and exacerbation phenotypes. The findings of these studies show that molecular microbiome technology has made significant advances in understanding the contribution of microbes to the pathogenesis and progression of COPD during both stable and exacerbation states^8–12^. However, little is known about the composition and clinical implications of the lung microbiome in hospitalized patients with COPD, especially for those with COPD and CAP requiring IMV and admission to the intensive care unit (ICU), who are considered to have the worst COPD outcomes.

The authors hypothesized that determining the changes in lung microbial composition could provide useful information for the management of patients with COPD and CAP requiring IMV and ICU admission. Therefore, the aims of the current study were to explore the longitudinal profiling of airway microbial composition, and compare the microbial changes over time between patients with COPD and CAP requiring IMV and admission to an ICU who had different weaning outcomes.

## Results

### Basic information

A total of 21 patients were included in the final analysis, 9 who had endotracheal aspirate samples from days 1, 7 and 14 of their respiratory ICU (RICU) hospitalization, and 12 who only had samples from days 1 and 7 (Supplementary Figure S1 online). Supplementary Table S1 online shows that all patients were elderly men who had a positive history of cigarette smoking and use of systemic corticosteroids and intravenous antibiotics during the study period. The baseline characteristics were similar between the patients with failed weaning and those with successful weaning.

### Taxonomy distribution

Rigorous quality checking and preprocessing removed about 20% of the reads, and those remaining were normalized to a total of 2,803,196 reads and 1,010,346 operational taxonomic units (OTUs), with a mean of 54,965 reads and 19,811 OTUs per endotracheal aspirate sample. For the saline samples there were a total of 27,191 reads and 10,622 OTUs, with a mean of 5,438 reads and 2,124 OTUs per sample.

**Figure 1:**
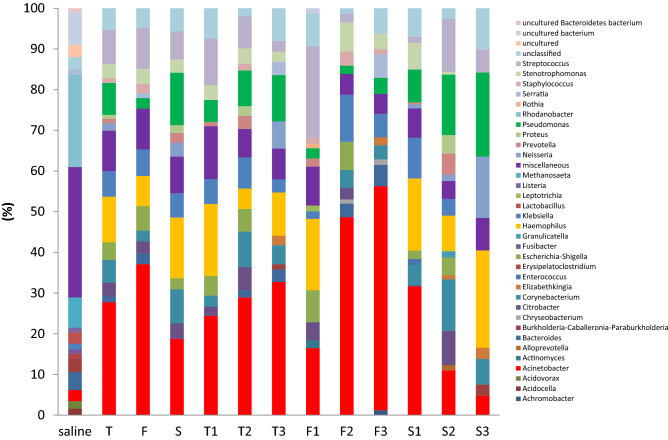
Comparisons of the bacterial community at the genus level by study group and sampling time points within individual study groups. 1, 2 and 3 represent days 1, 7 and 14 of respiratory intensive care hospitalization, respectively. *T* total participants, *F* failed weaning, *S* successful weaning.

Sequence analysis of the 51 samples from the participants and the 5 control samples revealed 728 genera representing 50 distinct phyla and 278 genera representing 31 distinct phyla, respectively. Supplementary Figure S2 online and Fig. 1 present the relative bacterial abundance of different phylogenetic levels stratified by study group and sampling time point. It shows that *Proteobacteria* was the most abundant bacterial phyla at any sampling time point across all study groups (Supplementary Figure S2 online) and there was no significant difference observed between all participants and controls (Supplementary Figure S3a online) or between sampling time points within individual study groups (Supplementary Figure S4 online). An upward trend in the abundance of bacteria from the *Acinetobacter* genera was observed over time in patients with failed weaning but not in those with successful weaning (Fig. 1).

### Changes in the relative abundance

**Figure 2:**
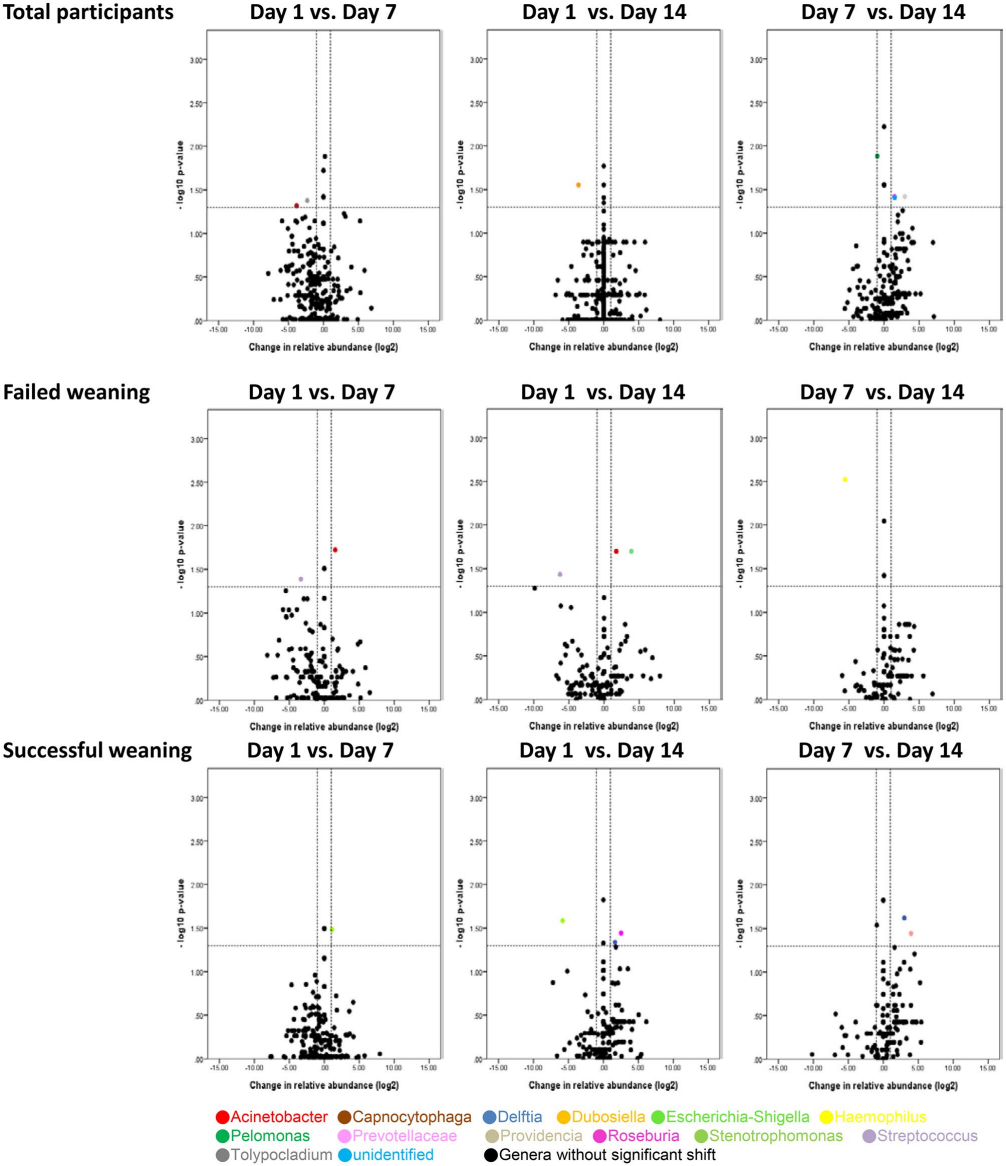
Temporal changes in the relative abundance of taxa by genus for all participants, patients with failed weaning and patients with successful weaning. Taxa that were significantly increased (upper right quadrant) or decreased (upper left quadrant) in pairwise comparisons of sampling time points are colored in the volcano plot. *vs.* versus.

**Figure 3:**
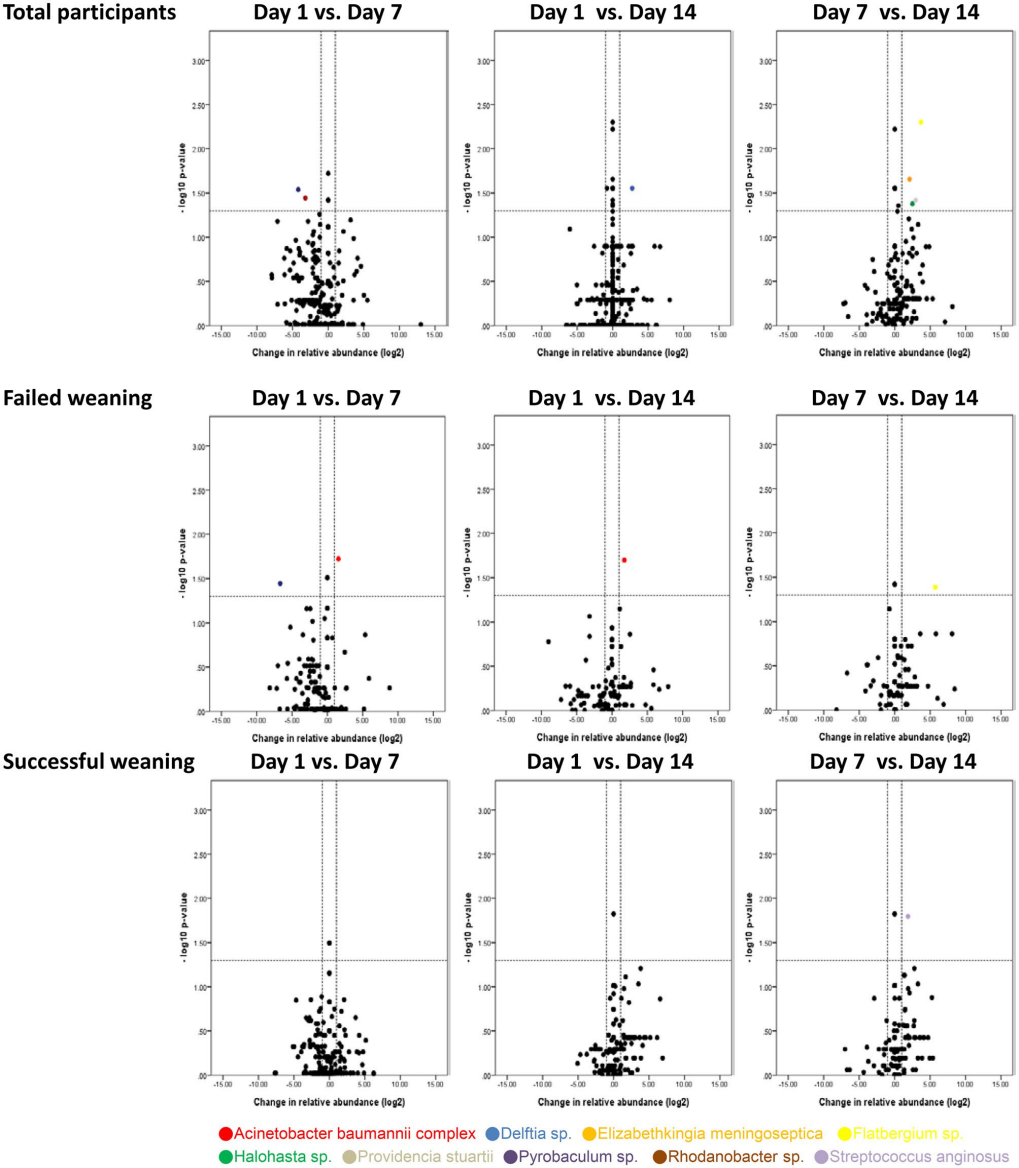
Temporal changes in the relative abundance of taxa by species for all participants, patients with failed weaning and patients with successful weaning. Taxa that were significantly increased (upper right quadrant) or decreased (upper left quadrant) in the pairwise comparisons of sampling time points are colored in the volcano plot. *vs.* versus.

Volcano plots show the taxa that had significant changes in their relative abundance (≥ twofold, *p* < 0.05) in paired comparisons between the study groups (patients vs. controls) and the sampling time points (day 1 vs. day 7; day 1 vs. day 14; day 7 vs. day 14) within each study group. Compared with the control group, significant changes in the relative abundance of many taxa at phylum, genus and species levels were observed in the patient group (Supplementary Figure S3a–S3c online). Meanwhile, several taxa at different phylogenetic levels had significant changes in their relative abundance when compared between the different sampling time points within individual study groups, although it was more apparent in patients with failed weaning than in those with successful weaning (Supplementary Figure S4 online and Figs. 2, 3). In particular, significant increases in the relative abundance of the *Acinetobacter* bacterial genus were observed between day 1 and day 7 and between day 1 and day 14 in patients with failed weaning but not in those with successful weaning (Fig. 2). In addition, the primary species from the *Acinetobacter* genus that demonstrated significant increases in their relative abundance was *A. baumannii* complex (Fig. 3).

**Figure 4:**
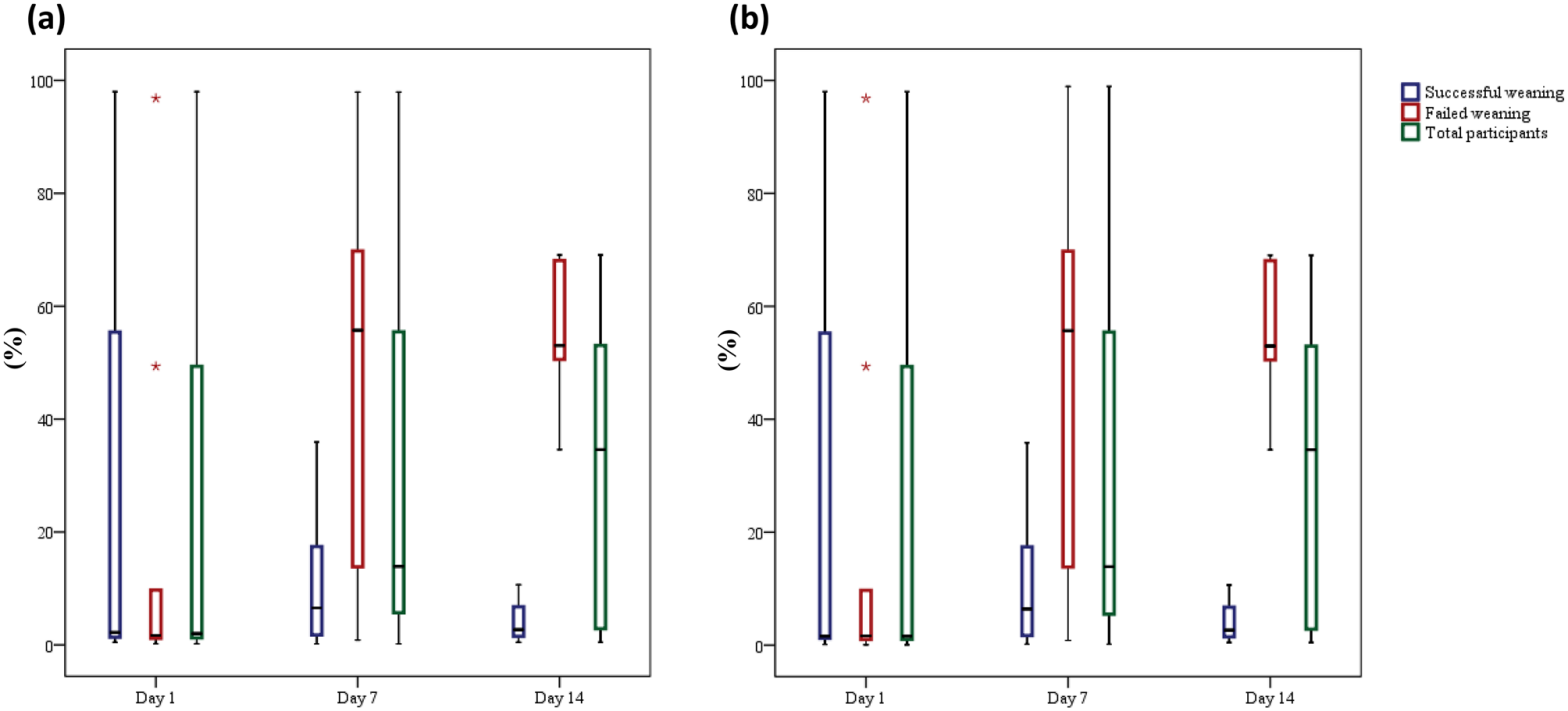
Changes over time in the relative abundance of (**a**) *Acinetobacter* genus, the *p* values for trend were 0.151, 0.012 and 0.335 for all participants, patients with failed weaning, and patients with successful weaning, respectively; and (**b**) *Acinetobacter Baumannii* complex species, the *p*-values for trend were 0.113, 0.012 and 0.426 for all participants, patients with failed weaning, and patients with successful weaning, respectively.

As the changes in the relative abundance of the *Acinetobacter* genus and the *A. baumannii* complex species were quite different between patients with different weaning outcomes (Figs. 2, 3), trend analyses were performed for the relative abundance of taxa for all participants, the patients with failed weaning and those with successful weaning. This revealed that the relative abundance of the *Acinetobacter* genus and the *A. baumannii* complex species did not show a significant upward or downward trend over time for all of the participants (p for trend = 0.151 and 0.113, respectively) and the patients with successful weaning (p for trend = 0.335 and 0.426, respectively). However, a significant increase in the relative abundance of the *Acinetobacter* genus (p for trend = 0.012) and the *A. baumannii* complex species (p for trend = 0.012) was observed in patients with failed weaning during the course of the study (Fig. 4a–b). These findings demonstrate that the pattern of changes in the relative abundance of the *Acinetobacter* genus and the changes in the *A. baumannii* complex species, were quite divergent between patients with different weaning outcomes.

### Quantitative polymerase chain reaction and sequencing validation studies

**Figure 5:**
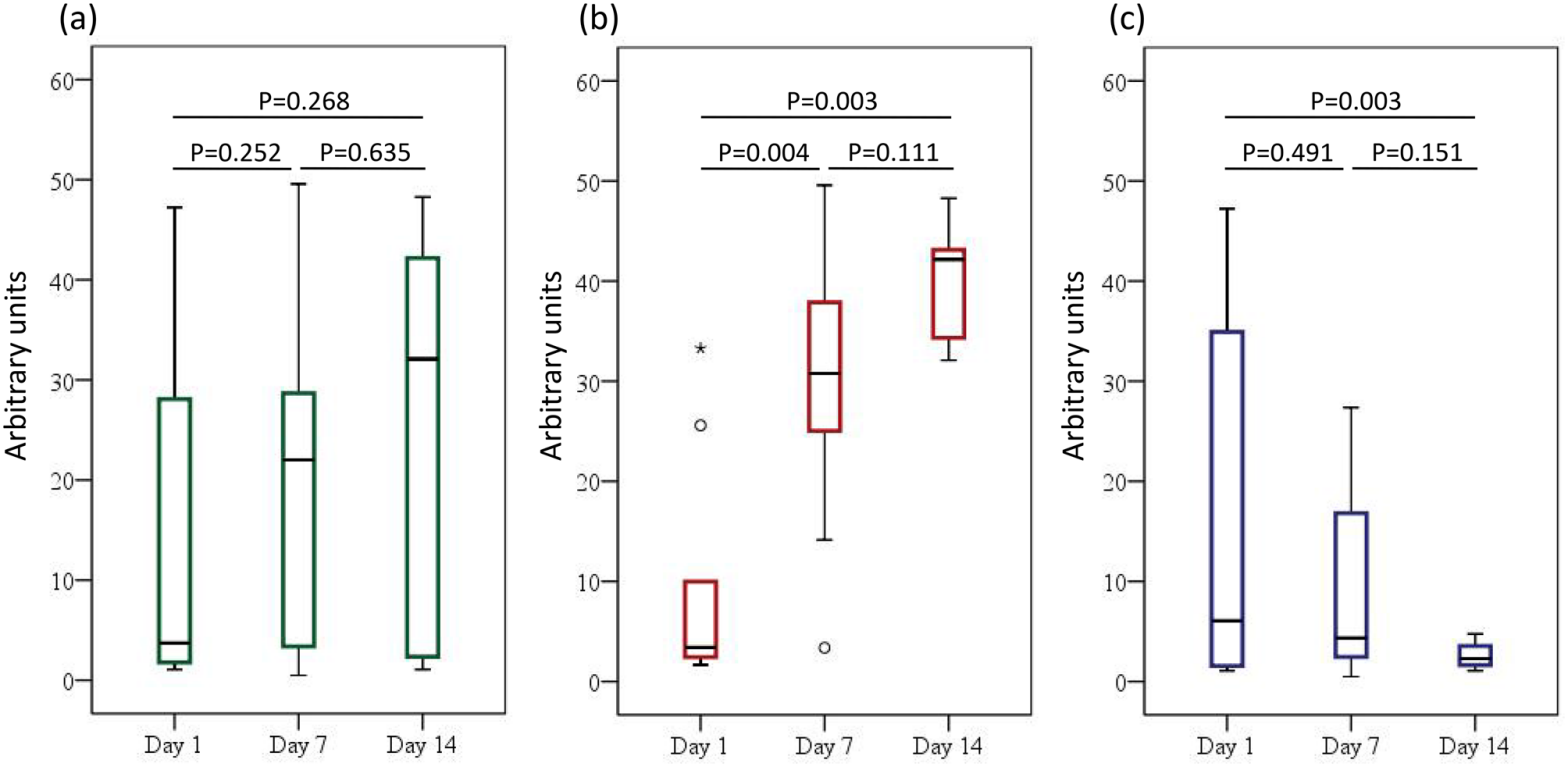
Quantitative polymerase chain reaction for *Acinetobacter baumannii* complex species for (**a**) total participants, (**b**) patients with failed weaning, and (**c**) those with successful weaning. The *p* values for trend were 0.156, 0.000, and 0.141 for all of the participants, patients with failed weaning, and those with successful weaning, respectively.

Figure 5 shows a sequence validation study with species-specific quantitative polymerase chain reaction (qPCR) for *A. baumannii* complex. It was found that the results were similar to those of the relative abundance of the *A. baumannii* complex species as shown in Figs. 3 and 4b.

### Changes in the alpha diversity

The alpha diversity, as determined by the Shannon diversity index, the observed species index and the Chao1 index, did not differ significantly over time or in paired comparisons of sampling time points for all of the participants, patients with failed weaning and those with successful weaning. However, the Shannon diversity index (*p* = 0.001) and the Chao1 index (*p* = 0.001) were significantly decreased in patients compared with the controls (Supplementary Figure S5–S8 online).

### Comparisons of the composition of bacterial communities

**Figure 6:**
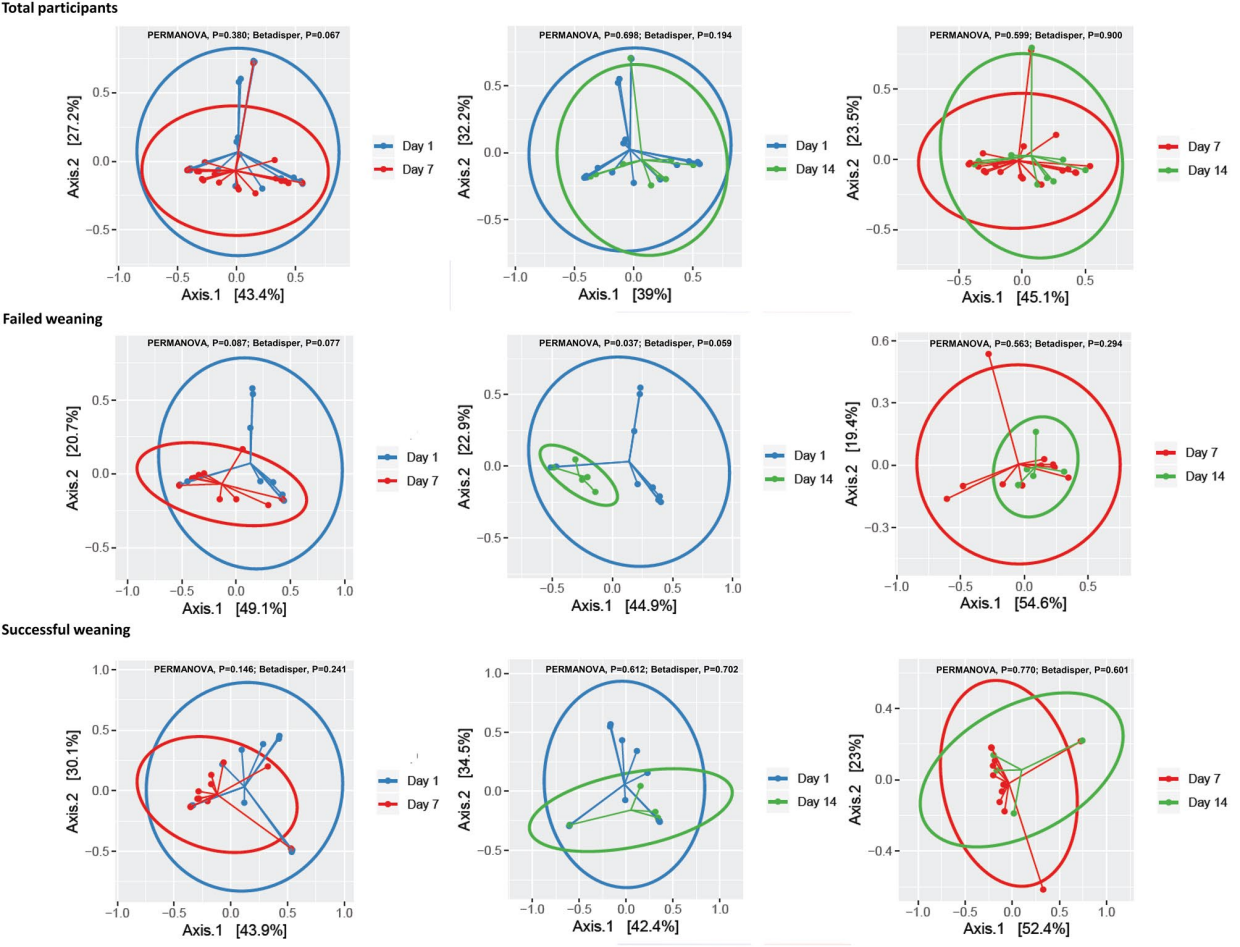
Differences in the composition of the bacterial community across paired comparisons of sampling time points within individual study groups.

The compositions of bacterial communities were significantly different between all of the participants and controls (PERMANOVA, *P* < 0.001; Betadisper, *P* = 0.356) (Supplementary Figure S9 online). Paired comparisons of the sampling time points showed no significant differences in microbial community structure in all of the participants and those with successful weaning. However, in patients with failed weaning differences were noted at days 7 and 14 of RICU hospitalization when compared with day 1 of the RICU hospitalization (PERMANOVA, *P* = 0.087; Betadisper, *P* = 0.077 and PERMANOVA, *P* = 0.037; Betadisper, *P* = 0.059, respectively) (Fig. 6). This suggests that temporal changes in the structure of the bacterial community vary between patients with different weaning outcomes. Together with the significant changes in the patterns of relative abundance of the *Acinetobacter* genus and the *A. baumannii* complex species in patients with failed weaning compared with successful weaning, these findings suggest that the invasion of this genus and species in the microbial community may be partly responsible for the change in microbiome structure in patients with failed weaning.

### Analyses of culture-dependent versus culture-independent sequencing for identification of the *A. baumannii* complex species

Supplementary Table S2 online shows that the discovery of the *A. baumannii* complex on conventional cultures did not guarantee the high relative species abundance of *A. baumannii* complex by sequencing and vice versa.

## Discussion

To the best of our knowledge the current study is the first to assess longitudinal changes in the microbial composition of the lungs of patients with COPD and CAP, requiring IMV and admission to an ICU. A key finding was that over time, those with failed weaning had a significant increase in the relative abundance of the *Acinetobacter* genus and the *A. baumannii* complex species, compared with patients with successful weaning, as well as a clear temporal change in the composition of their bacterial communities. This indicates that the invasion of this genus and species in the microbial community, at least in part, contributes to the shift in bacterial community composition in such patients.

The impact of *A. baumannii* complex infection, as identified by traditional culture methods, on adverse outcomes in critically ill patients is currently under debate. Several studies have reported that *A. baumannii *infections are associated with increased mortality in critically ill patients, while Huang et al*.* revealed that the identification of *A. baumannii* complex in endotracheal aspirate cultures from critically ill patients with COPD and CAP was not associated with adverse outcomes, in terms of prolonged length of ICU stay, failed weaning, death and readmission within 3 months^13–15^. The present study found that changes in lung microbiome structure had different features between patients with different weaning outcomes. Patients with failed weaning had an increase in the relative abundance of the *Acinetobacter* genus and the *A. baumannii* complex species over time in paired comparisons of sampling time points, and there was a significant difference in the composition of the bacterial communities between sampling time points compared with patients with successful weaning. Although further large-scale, well-designed studies are needed to validate these findings, the present study indicates that longitudinal analysis of the airway microbiome, particularly regarding the *Acinetobacter* genus and *A. baumannii* complex species, may offer useful predictive information on clinical outcomes in critically ill patients. Furthermore, the current findings also raise the question of whether the invasion of *A. baumannii* complex is secondary to a lack of judicious antibiotic use and emphasizes the need for infection control measures, which aim to prevent the transmission of *A. baumannii* complex.

Previous longitudinal airway microbiome studies in patients with COPD, have found an increase in the relative abundance of *Proteobacteria* at the onset of exacerbations compared with the clinically stable period^8,11^. Furthermore, during the stable state, patients with more severe COPD have been shown to have more abundant *Proteobacteria*^12,16,17^. The results of the current study, revealed that levels of *Proteobacteria* did not differ significantly over time within the individual study groups. These findings indicate that changes in the relative abundance of *Proteobacteria*, which contains a wide variety of genus and species, may depend on the disease state of COPD.

Via longitudinal analysis of sputum samples, previous microbiome studies have indicated that fluctuations in several bacterial OTUs, including *Moraxella*, *Streptococcus* and *Haemophilus* genera, could greatly impact the overall structure of microbial communities during COPD exacerbations and between patients with COPD of different severities^11,12,18^. However, the present study found that significant increases in the *Acinetobacter* genus were partly responsible for the changes in microbial community structure, which were seen in patients with COPD and CAP requiring IMV and ICU admission who had failed weaning. These results suggest that diverse OTUs contribute to changes in microbiome structure in patients with COPD in different disease states.

Unlike the present finding that there was no significant difference in alpha diversity over time or in paired comparisons of sampling time points for all study groups, a reduced alpha diversity has been previously reported during exacerbations compared with stable states in patients with COPD^11,12^. Together with the findings that there are differing changes in the relative abundance of *Proteobacteria* in varying COPD disease states, and that diverse genera contribute to the microbiome changes in different disease states, it has been suggested that the stability of the lung microbiome decreases with different disease states in COPD, and that a complex association exists between the disease state and microbiome structure.

Evidences indicated that, bacteria were more frequently discovered in the bronchoalveolar lavage fluid specimens via 16S rRNA gene sequencing compared to the culture-dependent techniques while the conventional culture combined with matrix assisted laser desorption ionization-time of flight mass spectrometry may be more sensitive for detecting certain bacteria^19,20^. Together with our findings that there was not a relationship existing between the presence of *A. baumannii* complex on the conventional culture and the relative bacterial abundance of *A. baumannii* complex species via the sequence analysis, the next generation sequencing may be complementary to the culture-dependent technique in microbial identification.

The strengths of the current study included the sample collection from endotracheal aspirates, the longitudinal nature of the study, and the unique study population that to the best of our knowledge, has not been previously evaluated in the research of airway microbiomes. This means that temporal samples with less oropharyngeal contamination were included, which provided a dynamic framework for the microbiome for the first time in this population. These features compensate for several limitations of the study, including that the microbiome analysis was only performed on 51 samples derived from a small number of participants who received corticosteroid and antibiotic treatments that may have effects on the lung microbial composition^8,11^. Furthermore, the potential association between the finding that there was a significant change in the relative abundance of the *Acinetobacter* genus and the *A. baumannii* complex species, and the obvious temporal difference in bacterial community composition between patients with failed weaning and those with successful weaning, could not be evaluated due to the study design. Further well-designed studies enrolling a larger number of participants are necessary to make lung microbiome analyses more applicable to clinical practice.

Though a one patient case study showed that the longitudinal dynamics in microbiota using induced sputum samples from six stable state visits and seven exacerbations for analysis was not related to disease states of COPD, the present longitudinal airway microbiome study determined that there were significantly different lung microbiome compositions between patients with different weaning outcomes^21^. Furthermore, one previous study found that two clusters differentiated based on dominance of *Haemophilus* were identified by sputum microbiota analysis in stable state patients with severe asthma and moderate-to-severe COPD^22^. Taken together, this advances the understanding of how microbiome composition analysis may be used within the clinic and reminds physicians that judicious antibiotic treatment and adequate infection control measures are important, which may have an impact on the clinical outcomes of patients with airway diseases. Larger studies should be performed to investigate whether specific microbiome compositions and functional determinants could be useful indicators for short-, medium- and long-term outcomes in patients with COPD and asthma.

## Conclusions

The findings of the current study provide useful information that increases the awareness of researchers and clinicians with regards to the clinical application of longitudinal airway microbiome structure analysis in the management of critically ill patients with and without COPD.

## Methods

### Study design and population

The current prospective, longitudinal, observational study was conducted at the RICU of Taichung Veterans General Hospital (TCVGH). It included patients with COPD and CAP requiring endotracheal intubation and IMV. The Institutional Review Board and Ethics Committee of TCVGH approved this study (approval number: CF13072), and confirmed that the research was performed in accordance with relevant guidelines/regulations. Informed consent was provided by the participants’ relatives or guardians. Further details are provided in Supplementary Methods S1 online.

### Data collection

The clinical features of each participant were collected throughout the study and the patient’s hospitalization in the RICU (see Supplementary Methods S2 online for further details).

### Sample collection and processing

For each participant, endotracheal aspirates were collected and processed according to standard methods for microbiome analyses; 16S rRNA gene sequencing of the genomic deoxyribonucleic acid (DNA) was performed and microbiological tests were conducted using conventional culture techniques, to identify potential bacterial respiratory pathogens at days 1, 7 and 14 (if the patients were still under endotracheal intubation) during their RICU hospitalization^23,24^. Patients with a time of endotracheal intubation < 7 days and therefore with only 1 endotracheal aspirate sample (day 1), were excluded from the study due to the study’s longitudinal design. In addition, 5 saline samples were collected and processed via the same methods used to collect the patients’ endotracheal aspirate samples as technical negative controls.

### Weaning process and outcomes

The weaning process and definitions of weaning outcomes are detailed in Supplementary Methods S3 online.

### Microbiota analysis

Genomic DNA was extracted from both endotracheal aspirate and saline samples for microbiota analysis. Further details are provided in Supplementary Methods S4 online.

### 16S quantitative polymerase chain reaction

*A. baumannii* complex species was quantified using qPCR for the endotracheal aspirate samples. The details are available in Supplementary Methods S6 online.

### Statistical analysis

All statistical analyses were performed using SPSS statistical software, version 18.0 (SPSS Inc., Chicago, IL, USA) and R (https://www.r-project.org). Statistical significance and a significant trend were set at *p* < 0.05. Further statistical information is detailed in Supplementary Methods S6 online.

## Supplementary information

Supplementary information

## Data Availability

The datasets generated and/or analyzed during the current study are available from the Sequence Read Archives database (accession number: PRJNA..541815. URL: https://dataview.ncbi.nlm.nih.gov/object/PRJNA541815?reviewer=raqv1op40l6je8633l6qc64guk.)
