# Supplementary material for: Dynamics of the lung microbiome in intensive care patients with chronic obstructive pulmonary disease and community-acquired pneumonia
Source: Sci Rep. 2020 Jul 6;10:11046. doi: 10.1038/s41598-020-68100-4 (PMC7338533; doi:10.1038/s41598-020-68100-4)
Supplement: Supplementary file 1 — Supplementary information [file 41598_2020_68100_MOESM1_ESM.pdf]

SUPPLEMENTARY INFORMATION

TITLE: Dynamics of the lung microbiome in intensive care patients with chronic obstructive pulmonary disease and community-acquired pneumonia

Authors' full names: Wei-Chang Huang MD, Ming-Feng Wu PhD, Chen-Cheng Huang MD, Sin-Yin Liu MD, Hui-Chen Chen MD, Yih-Yuan Chen PhD, Jeng-Yuan Hsu PhD, Chieh-Chen Huang PhD

Supplementary Methods S1- Study design and population

This prospective, longitudinal, observational study was conducted at the respiratory intensive care unit (RICU) of Taichung Veterans General Hospital, a tertiary teaching hospital in central Taiwan, between January 2014 and February 2016. Subjects who had previously been diagnosed with chronic obstructive pulmonary disease (COPD) based on the Global Initiative for Chronic Obstructive Lung Disease recommendation, were admitted to the RICU within 24 hours of arrival to the emergency department, and presented with community-acquired pneumonia (CAP) requiring endotracheal intubation and invasive mechanical ventilation (IMV) as decided by a physician were enrolled in this study.[1] Pneumonia was defined by clinical and radiological criteria and was considered to be CAP if it was acquired outside a hospital or nursing home and if the patient had not been hospitalized in the month prior to the development of pneumonia.[2,3] Patients who had a history of asthma, bronchiectasis, lung cancer, and other respiratory diseases were excluded from this study. In addition, those who were extubated within 7 days of the RICU hospitalization were excluded due to the longitudinal nature of our study design. Those who underwent tracheostomy or received antibiotic treatment for infections other than CAP during the study period were also excluded to avoid possible changes in airway microbiome composition and weaning outcomes due to these interventions.

Supplementary Methods S2- Data collection

The clinical features of each participant were collected throughout the study RICU hospitalization, including age, gender, smoking history, body mass index, and spirometry results within 2 years prior to this admission as performed and interpreted based on the American Thoracic Society statement.[4] The type of COPD pharmacological maintenance medication, other medications including systemic steroids and the type of antibiotic used

were also recorded. In addition, the modified Glasgow Coma Scale with verbal score as one,[5] previous history of admissions and use of antibiotics and systemic steroids within the 3 months prior to study entry were also recorded. The pneumonia severity index, which classifies patients with CAP into five ordered risk classes,[6,7] chest X-ray findings, initial laboratory findings and ventilator settings upon arrival at the RICU were recorded. Other data collected included Acute Physiology and Chronic Health Evaluation II score, co-morbidities, microbiological testing of endotracheal aspirates at the sampling time points of interest which were evaluated by the central laboratory of the study institute based on standard procedures,[8,9] RICU length of stay, and weaning outcomes. All patient information was anonymized and de-identified prior to analysis.

Supplementary Methods S3- Weaning process and outcomes

During the study period, the same intensivist team worked in the RICU where consistent protocol-driven ventilator weaning was applied and implemented according to the standards of the RICU at the study institute. The weaning process has been previously reported,[10] in which successful weaning was defined as liberation from IMV support on discharge from the RICU. Otherwise, the patients were defined as having failed weaning.[10]

Supplementary Methods S4 - Microbiota analysis

Genomic deoxyribonucleic acid (DNA) was extracted from both endotracheal aspirates and saline samples using a taco DNA/RNA Extraction Kit (GeneReach Biotechnology, Taiwan), and individually collected and stored at –80°C for further study.

The V3-V4 hypervariable region of the 16S rRNA gene was amplified using Illumina composite primers (341F: ′5-CCTACGGGNGGCWGCAG-3′ and 805R: ′5-GACTACHVGGGTATCTAATCC-3′) with barcodes.[11] All polymerase chain reactions

(PCRs) were performed in a 25μL volume with 0.2μL AccuPrime Taq DNA Polymerase, high fidelity (Thermo Fisher Scientific), 0.5μM forward and reverse primers, and about 1 ng template DNA. Thermal cycling was performed with an initial denaturation step at 94˚C for 2 min, followed by 30 cycles of 94˚C for 20 sec, 56˚C for 30 sec, and 68˚C for 60 sec, then a final elongation step at 72˚C for 5min.

Equal volumes of 1x loading buffer (containing SYB green) and PCR products were mixed and electrophoresis on 2% agarose gel was performed for detection. Samples with 1 main bright strip between 450-500 bp were chosen for further experiments. Then, the mixed PCR products were purified using a QIAquick Gel Extraction Kit (QIAGEN). Quantitative analysis of the DNA from the gel extraction was performed using a Qubit dsDNA HS assay kit (Thermo Fisher Scientific).

The fragments generated had single-stranded, ‘sticky’ ends. The next step, called end-repair, fills in these sticky ends to create blunt ends, ready for adaptor ligation. Adaptors are then bound to both the 5’ and the 3’ ends of the library fragments. They are specific to the sequencing platform, but ultimately all serve to enable in-platform clonal amplification, i.e. Illumina’s bridge amplification. The adaptors are designed to bind to the sequence-specific substrate, such as a patterned flow cell, and contain sequences to enable amplification, and can have barcodes for fragment identification. Finally, the library was sequenced on an Illumina Miseq platform, generating 300 bp paired-end reads. The library quality was assessed using the Qubit 2.0 Fluorometer (Thermo Scientific) and Agilent Bioanalyzer1000 system.

Sequenced reads were filtered for quality and processed using the latest version of bioinformatics software package “QIIME v1.80”. Operational taxonomic units were clustered from chimera-cleaned reads at a 97% identity threshold and assigned taxonomy through the SILVA-based (version 132) reference database.

Supplementary Methods S5 - 16S quantitative polymerase chain reaction

Acinetobacter baumannii complex species was quantified using a quantitative polymerase chain reaction using published primers and probes for the endotracheal aspirate samples. Reactions were performed on an ABI ViiA7 Real-time PCR System (Applied Biosystems). The thermal-protocol was as follows: 50℃ for 2 min, 95℃ for 10 min, followed by 40 cycles of 95℃ for 15 s, and 60℃ for 1 min. Readings were taken in single acquisition mode. The primers and probes consisted of the primer 327-F: TCC TAC GGG AGG CAG CAG T, the primer 794-R: GGA CTA CCA GGG TAT CTA ATC TT and the 16S specific probe FAM-CGTATTACCGCGGCTG-TAMRA. Samples were run in duplicate and at 1:20- and 1:40-fold dilutions.

Supplementary Methods S6- Statistical analysis

All data were expressed as the median and interquartile range or as a number (percentage). Comparisons were performed using the Mann-Whitney U test for continuous variables and chi-square test for categorical variables. A volcano plot was generated using SPSS to display taxa with large magnitude changes that were also statistically significant. The trend analysis of relative abundances of genus *Acinetobacter*, species *A. baumannii* complex, and alpha diversity over time was analyzed using the Jonckheere-Terpstra test. Alpha diversity of the samples was assessed using the Shannon index, observed species index, and Chao1 index. Species relative abundance data were analyzed using principal coordinate analysis, which was conducted with R package vegan based on Bray-Curtis distance. Between-group inertia percentages were evaluated using R package ade4 with Monte-Carlo test under 10,000 permutations to determine the p value of ordination. Sample groups were distinguished by

colors with 95% confidence ellipses based on the standard deviations of the average axis scores.

References

1. Global Initiative for Chronic Obstructive Lung Disease (GOLD). Global strategy for the diagnosis, management, and prevention of chronic obstructive pulmonary disease: updated 2014. https://fysio.dk/globalassets/documents/fafo/kliniske-retningslinjer/gold_report_2014_jan23.pdf (2014).

2. Mandell, L.A., et al. Infectious Diseases Society of America/American Thoracic Society consensus guidelines on the management of community-acquired pneumonia in adults. *Clin Infect Dis.* **44**, S27-72 (2007).

3. Leung, W.S., et al. Fulminant community-acquired Acinetobacter baumannii pneumonia as a distinct clinical syndrome. *Chest.* **129**, 102-109 (2006).

4. Lung function testing: selection of reference values and interpretative strategies. American Thoracic Society. *Am Rev Resp Dis.* **144**, 1202-1218 (1991).

5. Huang, W.C., et al. High-frequency chest wall oscillation in prolonged mechanical ventilation patients: a randomized controlled trial. *Clin Respir J.* **10**, 272-281 (2016).

6. Fine, M.J., et al. Comparison of a disease-specific and a generic severity of illness measure for patients with community-acquired pneumonia. *J Gen Intern Med.* **10**, 359-368 (1995).

7. Fine, M.J., Singer, D.E., Hanusa, B.H., Lave, J.R. & Kapoor, W.N. Validation of a pneumonia prognostic index using the MedisGroups comparative hospital database. *Am J. Med.* **94**, 153-159 (1993).

8. Wilson, M.L. General principles of specimen collection and handling. *Clin Infect Dis.* **22**, 766-777 (1996).

9. Ko, F.W., et al. Sputum bacteriology in patients with acute exacerbations of COPD in Hong Kong. *Respir Med.* **99**, 454-460 (2005).

10. Huang, W.C., et al. Clinical features, bacteriology of endotracheal aspirates and treatment outcomes of patients with chronic obstructive pulmonary disease and community-acquired pneumonia in an intensive care unit in Taiwan with an emphasis on eosinophilia versus non-eosinophilia: a retrospective case-control study. *BMJ Open.* **8**, e020341; 10.1136/bmjopen-2017-020341 (2018).

11. Nossa, C.W., et al. Design of 16S rRNA gene primers for 454 pyrosequencing of the human foregut microbiome. *World J Gastroenterol.* **16**, 4135-4144 (2010).

Supplementary Table S1. Baseline demographics and characteristics of the enrolled participants.

	Failed weaning (n=10)	Successful weaning (n=11)	Total (n=21)	p value
Age (years)	85.5 (79.3, 87.5)	82.0 (78.0, 86.0)	85.0 (79.5, 86.5)	0.619
Male gender	10 (100%)	11 (100%)	21 (100%)	NA
Smoking history				1.000
Ex-smoker	10 (100%)	10 (90.9%)	20 (95.2%)	
Current smoker	0 (0.0%)	1 (9.1%)	1 (4.8%)	
BMI	23.0 (19.2, 25.8)	21.6 (19.4, 28.5)	21.8 (19.4, 27.6)	0.698
Spirometry (post-bronchodilator test)				
FEV1/FVC (%)	61.0 (55.3, 65.3)	59.0 (43.0, 63.0)	60.0 (50.5, 63.0)	0.305
FEV1/predicted (%)	65.0 (55.3, 68.0)	56.0 (42.0, 58.0)	58.0 (43.5, 66.5)	0.057
GOLD severity				0.709
II	8 (80.0%)	7 (63.6%)	15 (71.4%)	
III	1 (10.0%)	2 (18.2%)	3 (14.3%)	
IV	1 (10.0%)	2 (18.2%)	3 (14.3%)	
Inhaled pharmacological therapy				0.555
ICS/LABA	8 (80.0%)	10 (90.9%)	18 (85.7%)	
LAMA alone	1 (10.0%)	0 (0.0%)	1 (4.8%)	
LABA alone	1 (10.0%)	1 (9.1%)	2 (9.5%)	
Use of systemic steroids upon RICU hospitalization	10 (100%)	11 (100%)	21 (100%)	NA
Pneumonia severity index				1.000
91-130	3 (30.0%)	4 (36.4%)	7 (33.3%)	
>130	7 (70.0%)	7 (63.6%)	14 (66.7%)	
Extent of pneumonia on chest X-ray				0.361
Unilateral	2 (20.0%)	5 (45.5%)	7 (33.3%)	
Bilateral	8 (80.0%)	6 (54.5%)	14 (66.7%)	
RICU length of stay (days)	14.5 (11.5, 21.8)	13.0 (11.0, 19.0)	13.0 (11.5, 20.0)	0.723
Endotracheal aspirate culture at day 1				0.168
Staphylococcus aureus	3 (30.0%)	0 (0.0%)	3 (14.3%)	

Acinetobacter baumannii complex	2 (20.0%)	1 (9.1%)	3 (14.3%)	
Klebsiella pneumoniae	2 (20.0%)	3 (27.3%)	5 (23.8%)	
Haemophilus influenzae	0 (0.0%)	2 (18.2%)	2 (9.5%)	
Pseudomonas aeruginosa	1 (10.0%)	0 (0.0%)	1 (4.8%)	
Normal mixed flora	2 (20.0%)	5 (45.5%)	7 (33.3%)	
Major antibiotics prescribed in the first week of RICU hospitalization[&]				0.857
Linezolid	1 (10.0%)	0 (0.0%)	1 (4.8%)	
Piperacillin/Tazobactam	3 (30.0%)	3 (27.3%)	6 (28.6%)	
Ertapenem	0 (0.0%)	1 (9.1%)	1 (4.8%)	
Imipenem/Cilastatin	1 (10.0%)	1 (9.1%)	2 (9.5%)	
Amoxicillin/Clavulanate	2 (20.0%)	1 (9.1%)	3 (14.3%)	
Doripenem	1 (10.0%)	1 (9.1%)	2 (9.5%)	
Ampicillin/Sulbactam	1 (10.0%)	3 (27.3%)	4 (19.0%)	
Ceftriaxone	1 (10.0%)	1 (9.1%)	2 (9.5%)	
Endotracheal aspirate culture at day 7				0.281
A. baumannii complex	3 (30.0%)	2 (18.2%)	5 (23.8%)	
K. pneumoniae	2 (20.0%)	0 (0.0%)	2 (9.5%)	
H. influenzae	0 (0.0%)	1 (9.1%)	1 (4.8%)	
P. aeruginosa	3 (30.0%)	1 (9.1%)	4 (19.0%)	
Stenotrophomonas maltophilia	0 (0.0%)	1 (9.1%)	1 (4.8%)	
Corynebacterium species	0 (0.0%)	1 (9.1%)	1 (4.8%)	
Normal mixed flora	2 (20.0%)	5 (45.5%)	7 (33.3%)	
Major antibiotics prescribed in the second week of RICU hospitalization[&]				0.428
Piperacillin/Tazobactam	1 (10.0%)	4 (36.4%)	5 (23.8%)	
Ertapenem	1 (10.0%)	1 (9.1%)	2 (9.5%)	
Amoxicillin/Clavulanate	1 (10.0%)	1 (9.1%)	2 (9.5%)	
Doripenem	1 (10.0%)	1 (9.1%)	2 (9.5%)	
Ampicillin/Sulbactam	1 (10.0%)	2 (18.2%)	3 (14.3%)	

Ceftriaxone	2 (20.0%)	0 (0.0%)	2 (9.5%)	
Tigecycline	2 (20.0%)	0 (0.0%)	2 (9.5%)	
Levofloxacin	0 (0.0%)	1 (9.1%)	1 (4.8%)	
Teicoplanin	1 (10.0%)	0 (0.0%)	1 (4.8%)	
Cefepime	0 (0.0%)	1 (9.1%)	1 (4.8%)	
Endotracheal aspirate culture at day 14				0.061
Available number	5 (50.0%)	4 (36.4%)	9 (42.9%)	
A. baumannii complex	1 (20.0%)	0 (0.0%)	1 (11.1%)	
K. pneumoniae	1 (20.0%)	0 (0.0%)	1 (11.1%)	
P. aeruginosa	2 (40.0%)	0 (0.0%)	2 (22.2%)	
Proteus mirabilis	1 (20.0%)	0 (0.0%)	1 (11.1%)	
Normal mixed flora	0 (0.0%)	4 (100%)	4 (44.4%)	
Prior admission within 3 months	5 (50.0%)	4 (36.4%)	9 (42.9%)	0.670
Prior use of antibiotics within 3 months	6 (60.0%)	5 (45.5%)	11 (52.4%)	0.670
Prior use of systemic steroids within 3 months	7 (70.0%)	4 (36.4%)	11 (52.4%)	0.198
APACHE II score	31.5 (21.8, 36.3)	27.0 (22.0, 33.0)	28.0 (22.0, 35.0)	0.481
Modified GCS score#	3.0 (3.0, 7.3)	4.0 (3.0, 8.0)	3 (3, 7)	0.703
Laboratory findings				
WBC ($x10^9$/L)	10.9 (6.4, 18.2)	10.3 (9.0, 16.7)	10.5 (8.1, 17.0)	1.000
Hemoglobin (g/dL)	9.2 (8.1, 10.6)	9.9 (9.1, 11.8)	9.6 (8.8, 11.5)	0.260
Platelet ($x10^9$/L)	179.5 (115.0, 222.3)	153.0 (111.0, 258.0)	177.0 (115.5, 230.5)	0.888
Creatinine (mg/dL)	2.1 (0.9, 3.0)	2.0 (0.7, 2.4)	2.0 (0.9, 2.6)	0.460
BUN (mg/dL)	27.0 (19.0, 64.0)	30.0 (17.0, 76.0)	30.0 (19.0, 68.0)	0.778
pH	7.4 (7.3, 7.4)	7.4 (7.3, 7.4)	7.4 (7.3, 7.4)	0.725
PaO2 (mmHg)	77.0 (56.6, 113.6)	85.5 (80.8, 134.5)	84.7 (76.6, 117.1)	0.139
PaO2/FiO2 ratio	199.8 (138.1, 322.5)	258.0 (211.8, 307.1)	253.0 (193.3, 310.8)	0.205
Ventilator mode				0.090
Volume control	10 (100%)	7 (63.6%)	17 (81.0%)	
Pressure control	0 (0.0%)	4 (36.4%)	4 (19.0%)	
Co-morbidity				

Diabetes mellitus	5 (50.0%)	1 (9.1%)	6 (28.6%)	0.063
Cerebrovascular accident	0 (0.0%)	3 (27.3%)	3 (14.3%)	0.214
Hypertension	4 (40.0%)	8 (72.7%)	12 (57.1%)	0.198
Coronary artery disease	2 (20.0%)	6 (54.5%)	8 (38.1%)	0.183
Congestive heart failure	0 (0.0%)	2 (18.2%)	2 (9.5%)	0.476
Arrhythmia	4 (40.0%)	3 (27.3%)	7 (33.3%)	0.659
Valvular heart disease	2 (20.0%)	2 (18.2%)	4 (19.0%)	1.000

Data are presented as median (Q1, Q3) or number (%).

[&]Major antibiotics were defined as an antimicrobial that was given for 4 days or more in a week.

[#]Modified GCS score, verbal score as one.

Abbreviations: APACHE II score, Acute Physiology and Chronic Health Evaluation II score; BMI, body mass index; BUN, blood urea nitrogen; FEV1, forced expiratory volume in one second; FiO2, fractional inspired oxygen; FVC, forced vital capacity; GCS, Glasgow Coma Scale; GOLD, Global Initiative for Chronic Obstructive Lung Disease; ICS, inhaled corticosteroid; LABA, long-acting beta-agonist; LAMA, *long*-acting muscarinic antagonist; NA, not applicable; PaO2, arterial oxygen partial pressure; RICU, respiratory intensive care unit; WBC, white blood count.

Supplementary Table S2. The individual results of conventional cultures and sequence analyses regarding the *Acinetobacter* genus and the *A. baumannii* complex species for all participants.

	Endotracheal aspirate conventional culture			Microbiota analysis numbers of total qualified reads/relative bacterial abundance of *Acinetobacter* genus (%) /relative bacterial abundance of *Acinetobacter baumannii* complex species (%)		
	Day 1	Day 7	Day 14	Day 1	Day 7	Day 14
**Patients with failed weaning**						
F-01	*Staphylococcus aureus*	*A. baumannii* complex	*Pseudomonas aeruginosa*	35,086/1.2/1.0	30,723/69.8/69.8	34,193/50.6/50.5
F-02	Normal mixed flora	*P. aeruginosa*	NA	33,558/1.2/1.0	35,573/0.8/0.8	NA
F-03	*A. baumannii* complex	*A. baumannii* complex	NA	55,427/96.8/96.8	50,483/97.9/97.9	NA
F-04	*Klebsiella pneumoniae*	*A. baumannii* complex	*P. aeruginosa*	59,223/0.8/0.8	65,293/58.5/58.5	49,534/53.1/53.0
F-05	*S. aureus*	*K. pneumoniae*	NA	27,486/2.0/2.0	36,513/84.6/84.6	NA
F-06	*S. aureus*	*K. pneumoniae*	*A. baumannii* complex	50,994/9.7/9.7	74,014/13.8/13.8	75,901/34.6/34.6
F-07	*K. pneumoniae*	*P. aeruginosa*	*K. pneumoniae*	64,332/0.2/0.1	64,908/39.5/39.5	31,574/68.1/68.1
F-08	*P. aeruginosa*	*P. aeruginosa*	NA	51,575/2.1/2.1	69,165/56.0/55.9	NA
F-09	Normal mixed flora	Normal mixed flora	NA	73,417/1.4/1.3	61,549/10.3/10.3	NA
F-10	*A. baumannii* complex	Normal mixed flora	*Proteus mirabilis*	56,208/49.4/49.4	96,608/55.5/55.4	65,214/69.1/69.0
**Patients with successful weaning**						

S-01	*A. baumannii* complex	Normal mixed flora	Normal mixed flora	33,806/60.3/60.1	46,015/5.7/5.5	53,691/2.9/2.8
S-02	Normal mixed flora	*Stenotrophomonas maltophilia*	NA	69,852/1.4/1.2	44,122/21.5/21.5	NA
S-03	*K. pneumoniae*	Normal mixed flora	NA	52,508/50.6/50.4	59,273/13.9/13.9	NA
S-04	*Haemophilus influenzae*	*H. influenzae*	Normal mixed flora	51,213/1.3/1.3	33,516/1.0/1.0	41,749/0.5/0.5
S-05	Normal mixed flora	Normal mixed flora	Normal mixed flora	35,296/34.6/34.2	64,503/35.9/35.9	96,546/10.7/10.6
S-06	Normal mixed flora	*A. baumannii* complex	Normal mixed flora	57,529/96.1/96.1	4,189/6.6/6.4	12,266/2.5/2.5
S-07	*K. pneumoniae*	Normal mixed flora	NA	36,001/2.2/0.1	69,083/0.4/0.3	NA
S-08	Normal mixed flora	*P. aeruginosa*	NA	12,354/1.2/1.2	16,927/2.5/2.4	NA
S-09	*K. pneumoniae*	*Corynebacterium* species	NA	53,600/1.6/1.6	27,530/0.2/0.2	NA
S-10	*H. influenzae*	Normal mixed flora	NA	91,608/0.5/0.5	102,064/20.9/20.9	NA
S-11	Normal mixed flora	*A. baumannii* complex	NA	78,502/98.0/98.0	31,606/12.2/12.2	NA

Abbreviations: NA, not applicable.

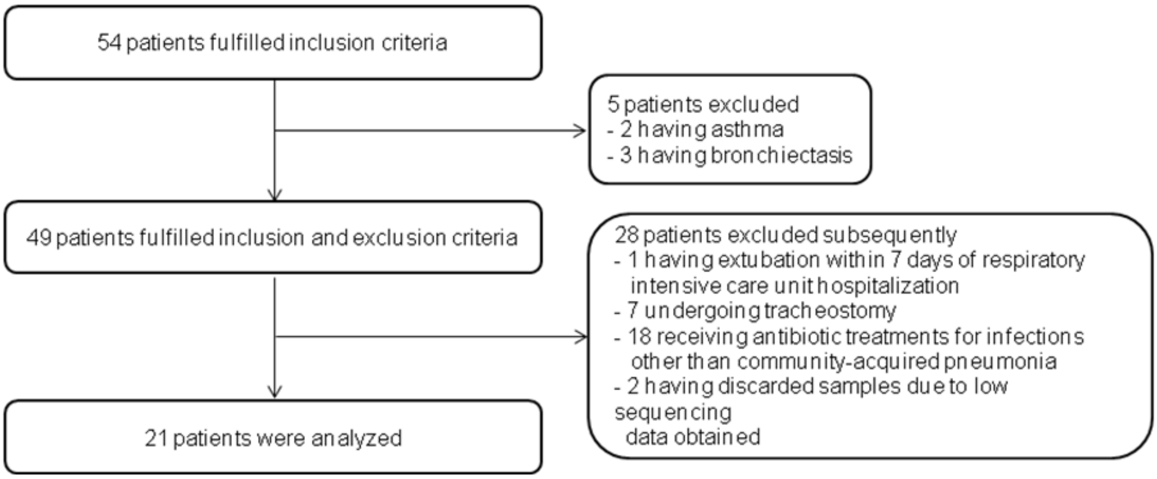

Supplementary Figure S1. The patient enrollment flow chart.

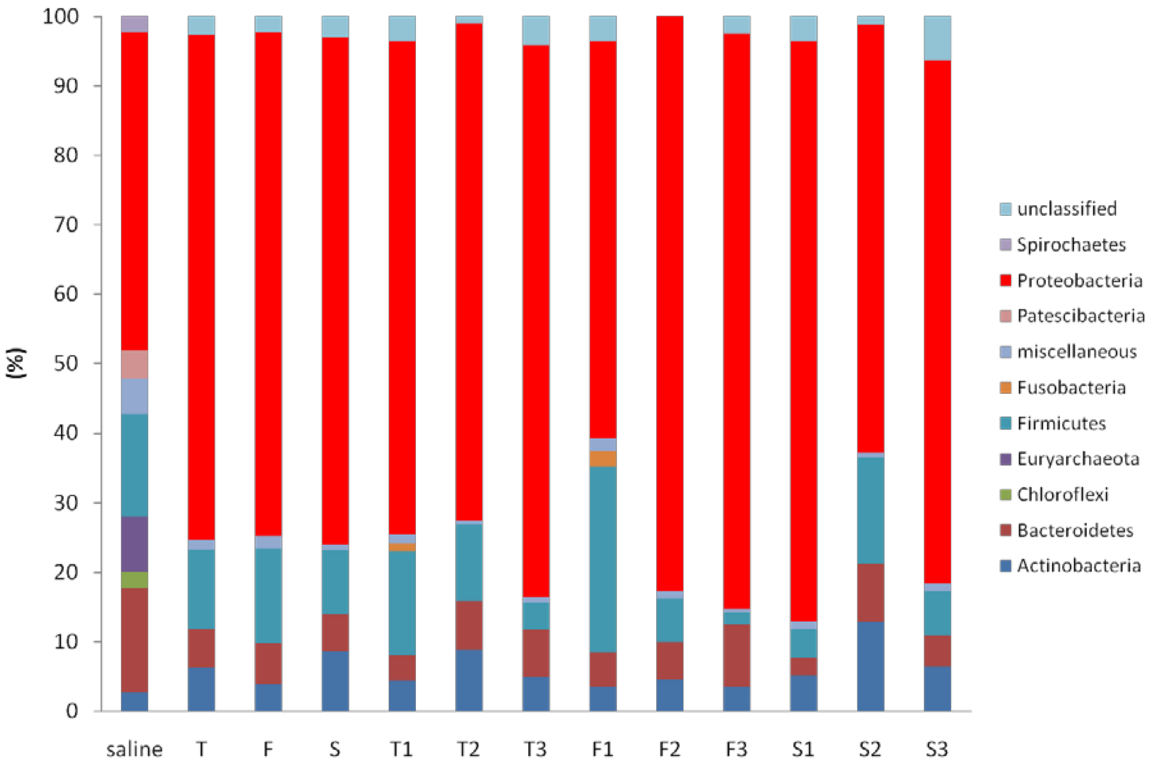

Supplementary Figure S2. Comparisons of the bacterial community at the phylum level by study group and sampling time points within individual study groups. 1, 2 and 3 represent days 1, 7 and 14 of respiratory intensive care hospitalization, respectively. Abbreviations: T, total participants; F, failed weaning; S, successful weaning.

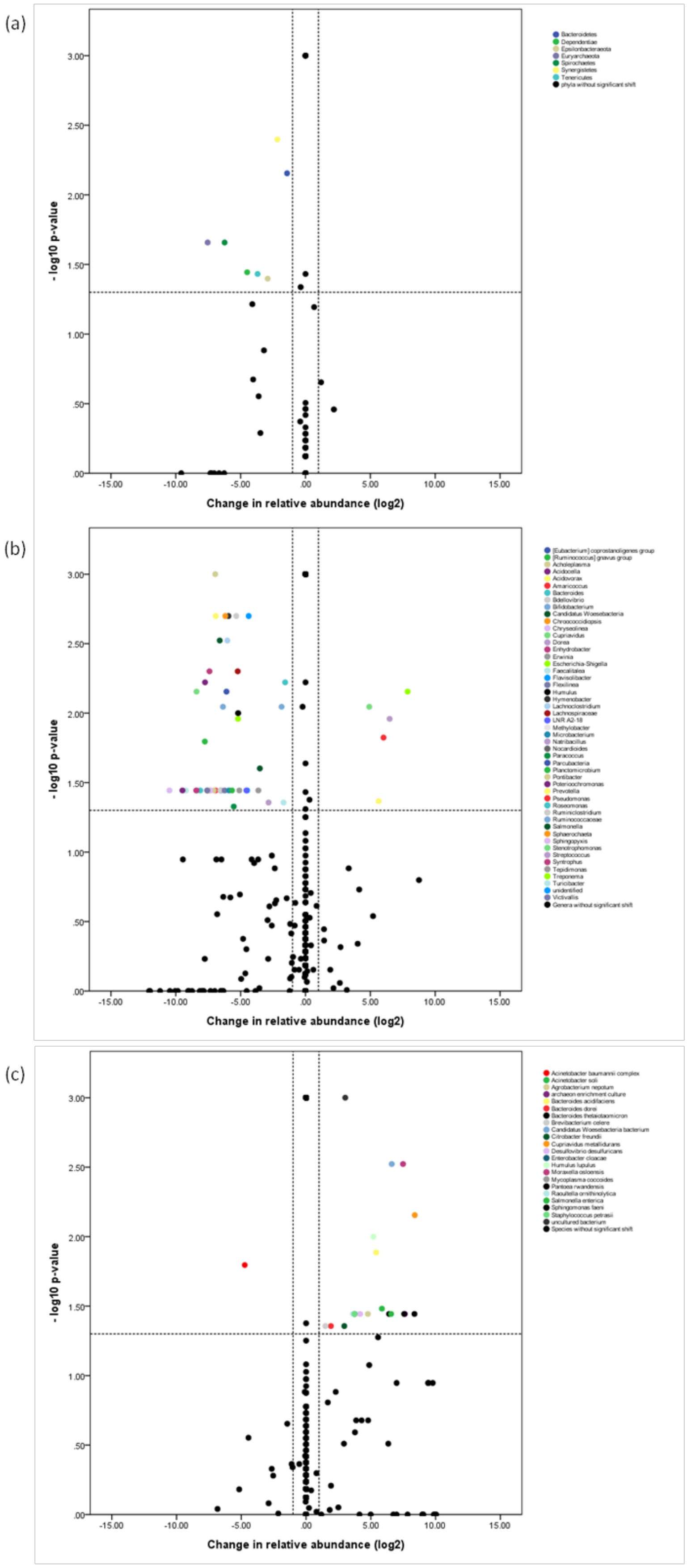
(a)
3.00
2.50
2.00
1.50
1.00
.50
.00
- log10 p-value
-15.00
-10.00
-5.00
.00
5.00
10.00
15.00
Change in relative abundance (log2)
Bacteroidetes
Dependentiae
Epsilonbacteraeota
Euryarchaeota
Spirochaetes
Synergistetes
Tenericutes
phyla without significant shift
(b)
- log10 p-value
Change in relative abundance (log2)
[Eubacterium] coprostanoligenes group
[Ruminococcus] gnavus group
Acholeplasma
Acidocella
Acidovorax
Amaricoccus
Bacteroides
Bdellovibrio
Bifidobacterium
Candidatus Woesebacteria
Chroococcidiopsis
Chryseolinea
Cupriavidus
Dorea
Enhydrobacter
Erwinia
Escherichia-Shigella
Faecalitalea
Flavisolibacter
Flexilinea
Humulus
Hymenobacter
Lachnoclostridium
Lachnospiraceae
LNR A2-18
Methylobacter
Microbacterium
Natribacillus
Nocardioides
Paracoccus
Parcubacteria
Planctomicrobium
Pontibacter
Poterioochromonas
Prevotella
Pseudomonas
Roseomonas
Ruminiclostridium
Ruminococcaceae
Salmonella
Sphaerochaeta
Sphingopyxis
Stenotrophomonas
Streptococcus
Syntrophus
Tepidimonas
Treponema
Turicibacter
unidentified
Victivallis
Genera without significant shift
(c)
- log10 p-value
Change in relative abundance (log2)
Acinetobacter baumannii complex
Acinetobacter soli
Agrobacterium nepotum
archaeon enrichment culture
Bacteroides acidifaciens
Bacteroides dorei
Bacteroides thetaiotaomicron
Brevibacterium celere
Candidatus Woesebacteria bacterium
Citrobacter freundii
Cupriavidus metallidurans
Desulfovibrio desulfuricans
Enterobacter cloacae
Humulus lupulus
Moraxella osloensis
Mycoplasma coccoides
Pantoea rwandensis
Raoultella ornithinolytica
Salmonella enterica
Sphingomonas faeni
Staphylococcus petrasii
uncultured bacterium
Species without significant shift

Supplementary Figure S3. Comparisons of the relative abundance of taxa by (a) phylum level (b) genus level and (c) species level between all of the participants and the controls. Taxa that were significantly increased (upper right quadrant) or decreased (upper left quadrant) in the comparisons are colored in the volcano plots.

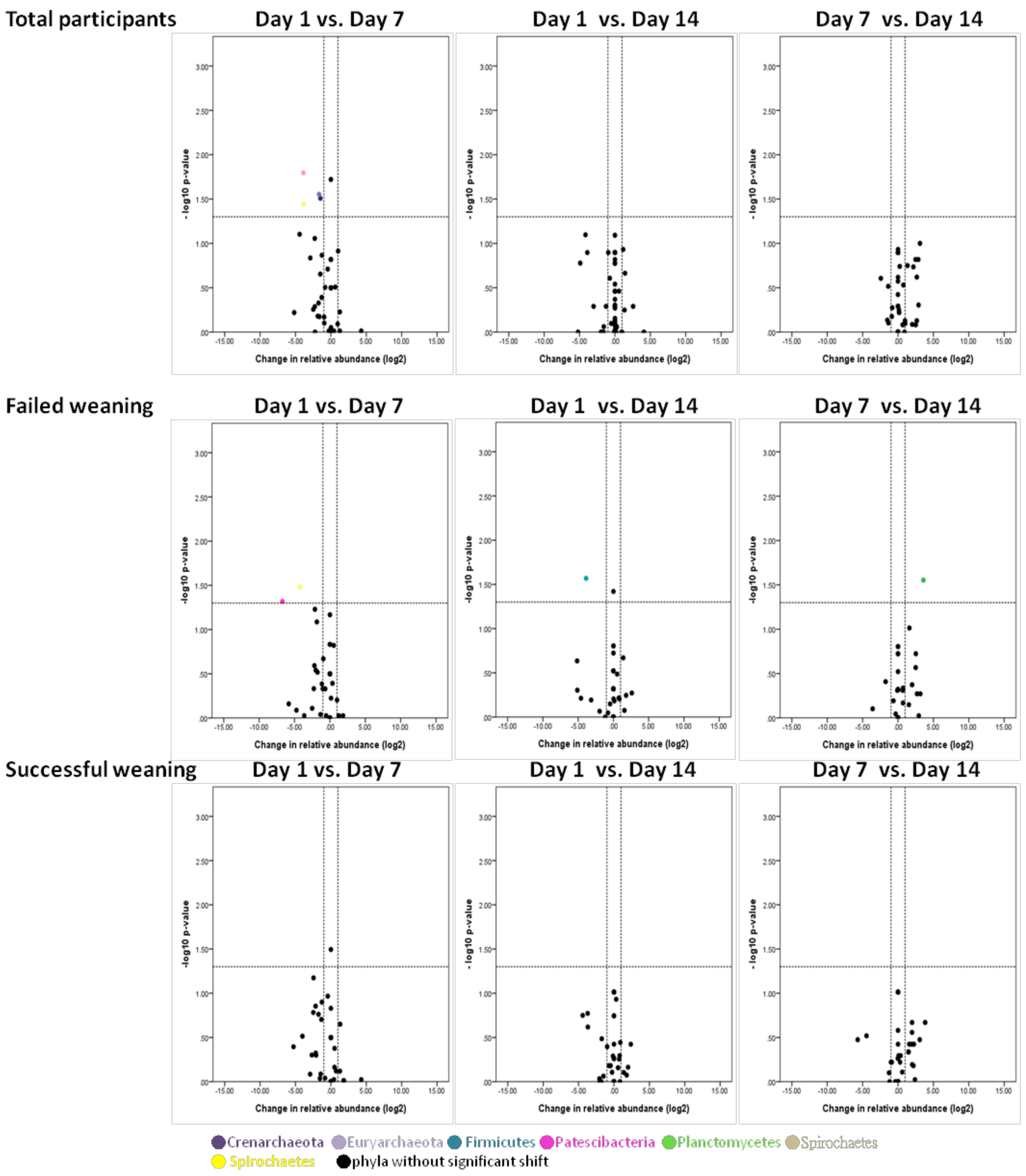

Supplementary Figure S4. Temporal changes in the relative abundance of taxa by phylum level for all of the participants, patients with failed weaning and those with successful weaning. Taxa that were significantly increased (upper right quadrant) or decreased (upper left quadrant) in the pairwise comparisons of sampling time points are colored in the volcano plot. Abbreviations: vs., versus.

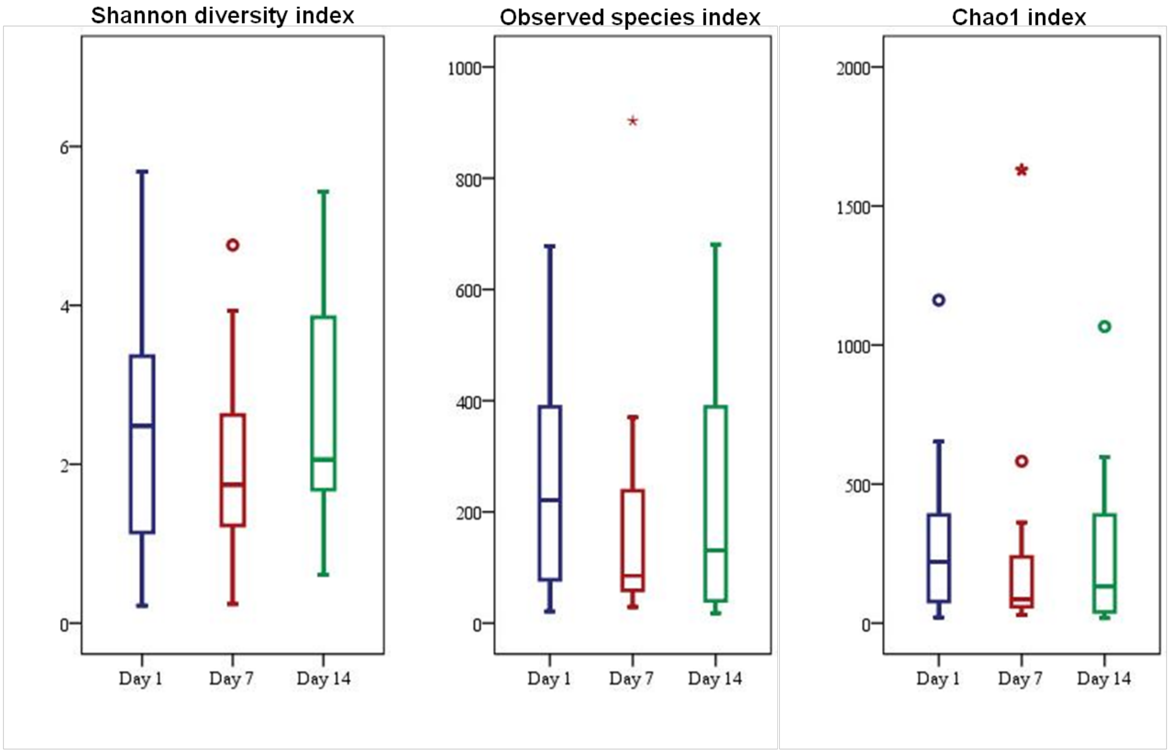

Supplementary Figure S5. Comparisons of temporal changes in alpha diversity for all of the participants. The p-values between day 1 and day 7, between day 1 and day 14, and between day 7 and day 14 for the Shannon diversity index, observed species index, and Chao1 index were 0.435, 0.700, and 0.354, 0.128, 0.657, and 0.717, 0.155, 0.625, and 0.751, respectively. The p-values for trend for the Shannon diversity index, observed species index, and Chao1 index were 0.972, 0.271, and 0.286, respectively.

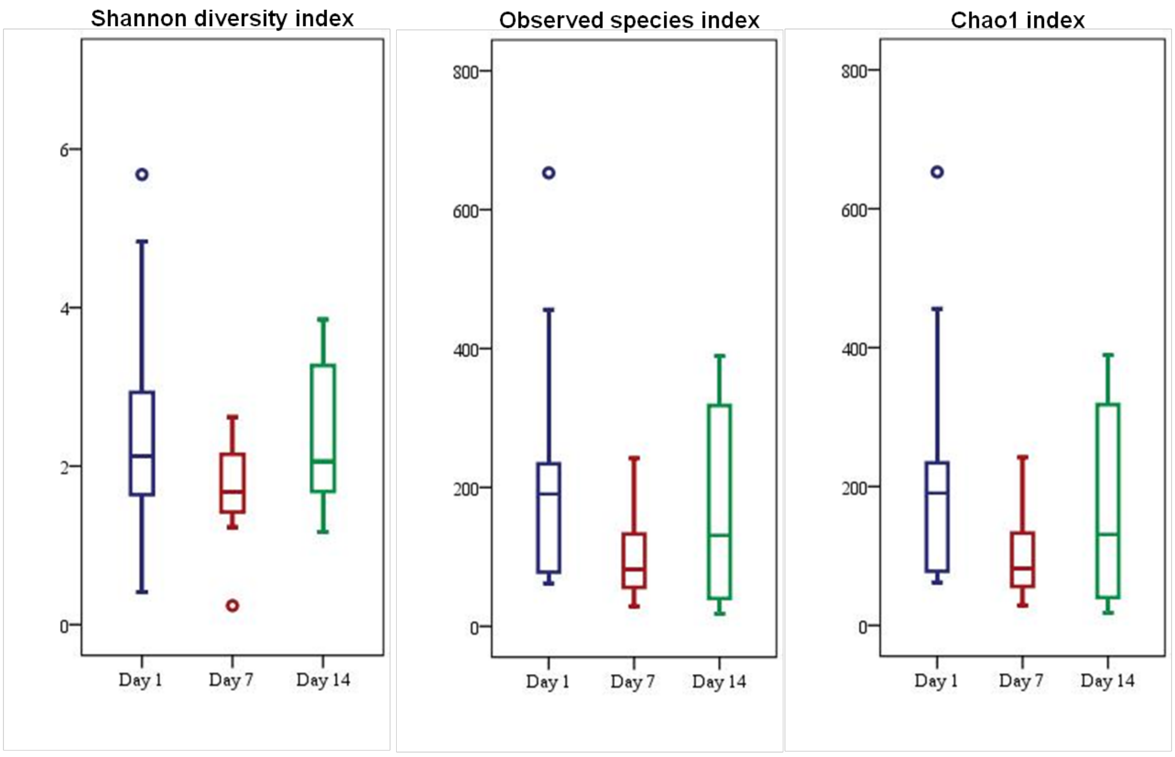

Supplementary Figure S6. Comparisons of temporal changes in alpha diversity for the patients with failed weaning. The *p*-values between day 1 and day 7, between day 1 and day 14, and between day 7 and day 14 for the Shannon diversity index, observed species index, and Chao1 index were 0.212, 0.903, and 0.327, 0.112, 0.540, and 0.624, 0.112, 0.540, and 0.624, respectively. The *p*-values for trend for the Shannon diversity index, observed species index, and Chao1 index were 0.705, 0.266, and 0.266, respectively.

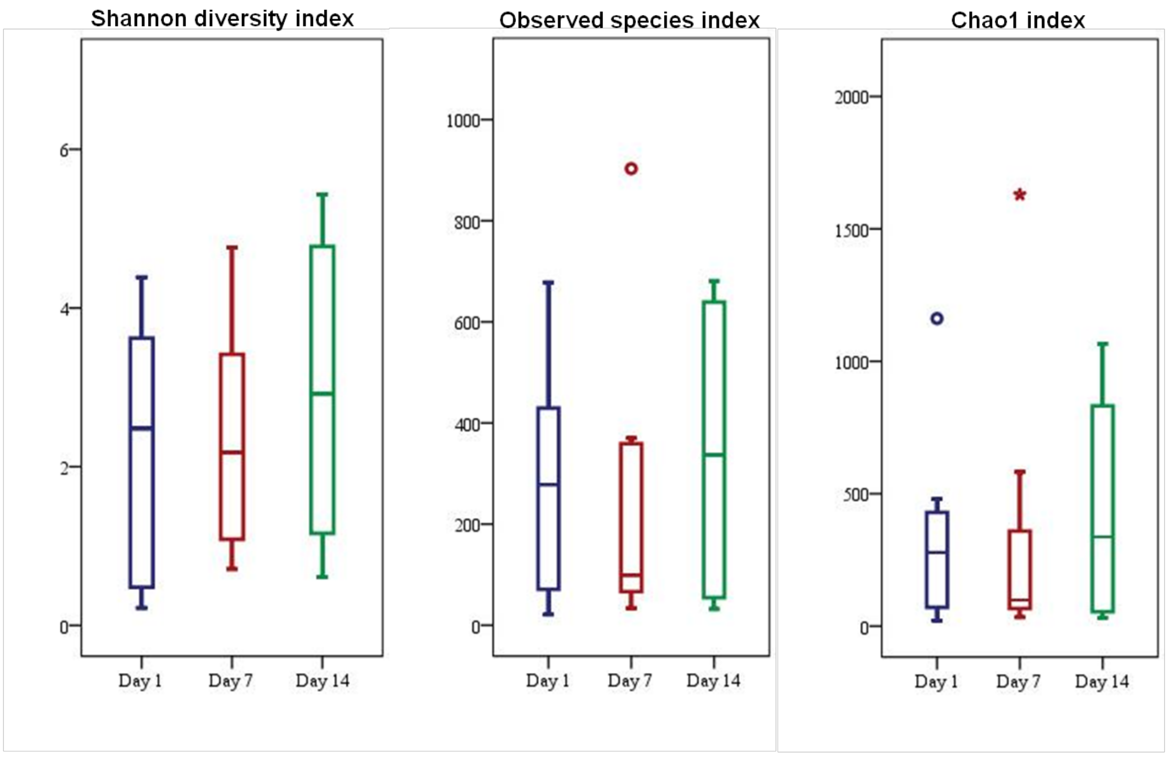

Supplementary Figure S7. Comparisons of temporal changes in alpha diversity for subjects with successful weaning. The *p*-values between day 1 and day 7, between day 1 and day 14, and between day 7 and day 14 for the Shannon diversity index, observed species index, and Chao1 index were 0.818, 0.361, and 0.602, 0.622, 0.695, and 0.794, 0.768, 0.794, and 0.794, respectively. The *p*-values for trend for the Shannon diversity index, observed species index, and Chao1 index were 0.484, 0.904, and 0.981, respectively.

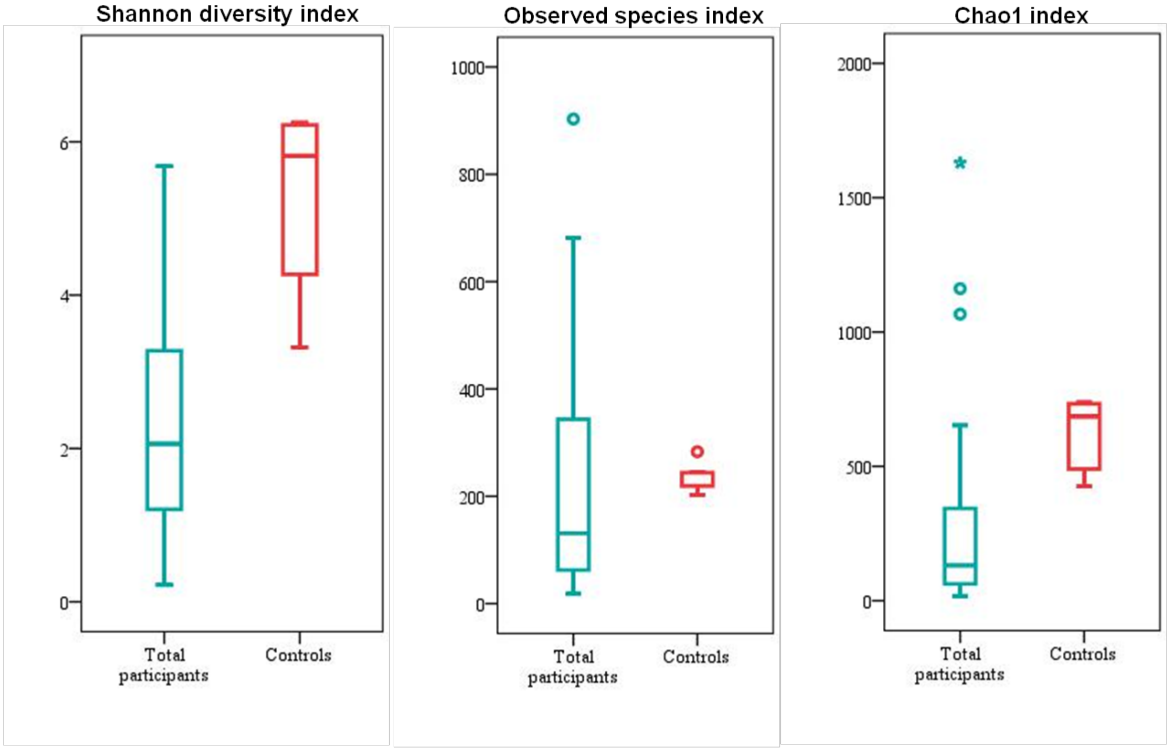

Supplementary Figure S8. Comparisons of alpha diversity between all of the participants and controls. The *p*-values for the Shannon diversity index, observed species index, and Chao1 index were 0.001, 0.354, and 0.001, respectively.

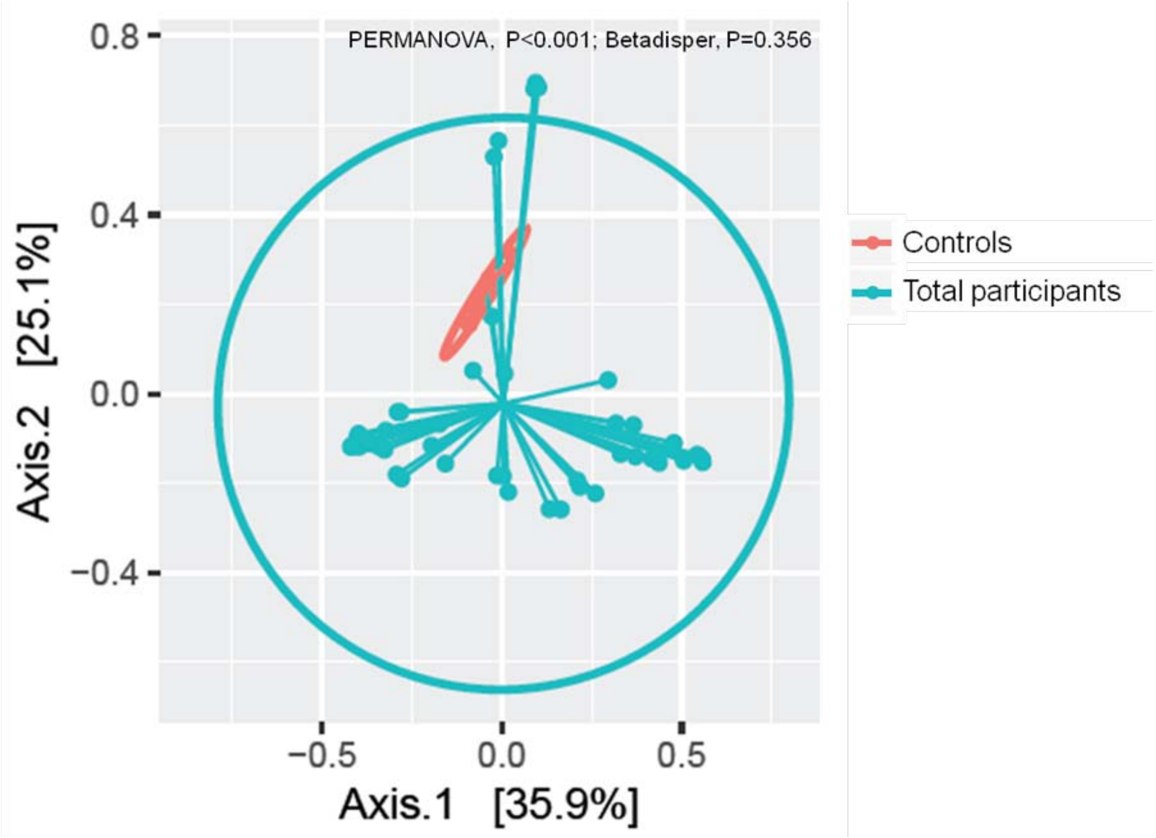

Supplementary Figure S9. Differences in bacterial community composition structure between all of the participants and controls.
